# EU’s Medical Device Expert Panels: Analysis of Membership and Published Clinical Evaluation Consultation Procedure (CECP) Results

**DOI:** 10.1007/s43441-024-00632-7

**Published:** 2024-06-10

**Authors:** Colleen Watson, Frances J. Richmond

**Affiliations:** Department of Regulatory and Quality Sciences, School of Pharmacy, University of Southern California, 1540 Alcazar Street, CHP 140, Los Angeles, CA 90089 USA

**Keywords:** Medical device, Medical device regulation, CECP, EMA, Expert panel

## Abstract

**Background:**

The new EU Medical Device Regulation (MDR) places greater importance on the role of clinical evidence to establish safety and performance. Article 54 of the MDR calls for expert committees to independently review the scientific, technical, and clinical evidence supporting the market authorization of certain novel devices independently from the established process of Notified Body reviews. These experts provide a review and opinion that ultimately is taken into consideration alongside the information reviewed by the Notified Body during the review process. Four expert committees (General and Plastic Surgery and Dentistry; Orthopaedics, Traumatology, Rehabilitation, Rheumatology; Circulatory System; and Neurology) have published at least one Scientific Opinion (SO) under the Clinical Evaluation Consultation Procedure (CECP) in 2021–2022.

**Methods:**

The four expert committees with published CECP opinions were reviewed to assess the academic backgrounds and professional expertise of each member with respect to clinical, technical, and biological domains on a 0–2 scale for each domain. A content review was conducted on the 10 CECP opinions published by these committees to assess their consistency with the goals and outcome expectations set by the MDR. The extent of content related to each of the clinical, technical, and biological domains was also assessed on a 0–2 scale.

**Results:**

All committees were composed primarily by members with strong clinical expertise, but only a few had strong technical and biological expertise. Across committees, the average scores of members related to academic background and professional expertise both ranged from 1.64 to 2.00 in the clinical domain, but only 0–0.15 and 0.15–0.69, respectively, in the biological domain, and 0.12–0.55 and 0.23–0.73, respectively, in the technical domain. A content review for the 10 SOs showed that all opinions focused exclusively or primarily on the clinical evidence. Three contained a modest amount of additional text directed at technical/engineering issues and five at biological issues.

**Conclusion:**

Expert committees are composed predominantly of expert clinical reviewers but have many fewer members with significant technical or biological expertise. This may limit the ability of the committees to evaluate the significant technical and biological risks that are often best understood by preclinical testing. Broadening the expertise across the committees may improve the depth of their benefit/risk critiques.

## Introduction

Regulatory agencies view patient safety as a primary focus when they evaluate whether medical devices are ready to market. To facilitate this oversight, the European Union (EU) introduced a new and more rigorous regulation, the Medical Device Regulation (EU 2017/745 [1]) (MDR). The regulation keeps the previously established EU device classification in which devices are tiered into class I, IIa, IIb, or III groupings according to escalating risks and complexity. It also preserves the core system of shared review, in which riskier class II and III devices submit manufacturing and testing information to a Notified Body, whose review is needed before applying a *Conformitè Europëenne (CE)* mark. The new MDR, however, places more demanding requirements on both the Notified Bodies that issue CE certification and the manufacturers that may have to perform additional testing or literature evaluation. This appears to have been driven at least in part by highly publicized problems with device safety and performance. These included, for example, patient injuries due to the substitution of industrial-grade silicone in poorly manufactured Poly Implant Prothese (PIP) breast implants and failure rates with metal hip implants resulting in considerable impact [2]. In the revised Regulations, a new step, called the Clinical Evaluation Consultation Procedure (CECP), under Article 54 of the MDR [1], was introduced for certain high-risk devices, such as implantables, active medical devices combined with medicinal substances, and high-risk novel devices. The CECP establishes expert panels, composed of technical, clinical, and scientific experts, who are responsible for critiquing the clinical performance and risk–benefit determinations of referred devices after the Notified Bodies have performed their analyses. These EC Expert Panels on Medical Devices and In Vitro Diagnostic Devices (EXPAMED) Committees do not replace the reviews of the Notified Bodies; EXPAMED experts provide a source for independent scientific analysis for Notified Bodies, the European Commission (EC), and manufacturers. Notified bodies may choose not to follow the recommendations of the CECP, but then must submit a full justification [3].

Initially, the EC assigned the development of the expert panels to the Joint Research Center (JRC), an independent arm of the EC that informs and develops methods and data to support EU policy. In 2019, JRC published a call for interest in these committees, directed at technical, clinical, and scientific experts who could analyze the clinical, performance and risk–benefit determinations for a device after the Notified Bodies have conducted their analyses. As stated on its website, “A key role for the experts will be to provide an independent opinion to Notified Bodies on the assessment of high-risk devices, before certification” [4, 5]. As opposed to Notified Body reviewers who undergo a training process to review specific types of devices and ultimately make the decision, EXPAMED members undergo a different qualification process. EXPAMED experts must have citizenship within the region, a university degree, 10 years of experience, English language, and no conflicting financial interest [5]. Then in January 2022, a new Regulation (EU) 2022/123 transferred oversight of these panels to the European Medical Agency (EMA) [6, 7]. As a consequence of this change, expert panels charged with reviewing device risk–benefit came under an agency with a primary focus on the oversight of drugs.

Although the EXPAMD experts may serve the EC in a variety of situations, in this study we examine the degree to which the committees are configured to accomplish their mandate defined by the relevant law and regulations through the Clinical Evaluation Consultation Procedure (CECP). Twelve (12) expert panels have been constituted to evaluate at different types of medical devices. Four of these, the neurology, orthopedics, circulatory, and general /plastic surgery/ dentistry panels, had published 10 scientific opinions (SOs) by February 2023 [8].

The committee memberships and publications of the 10 SOs now available for high-risk medical devices are of special interest because they may anticipate the types of outcomes and challenges that the new CECP system of secondary review may have going forward. To carry out this evaluation, we looked at the professional attributes of the committee members for the four (4) expert panels that have produced CECPs. We then conducted a content evaluation of the SOs to assess the scope and nature of issues that were addressed in their published opinions. We discuss whether the composition of the committees and the instructions that they are given are consistent with the original goals assigned to the expert panels by Article 54 and Annex IX section 5.1 of the MDR [1].

## Methods

### Member profiles

We examined the composition of four committees that had published SOs by April, 2023. To assess academic background and training, two researchers independently assessed the degrees, fellowships, and postdoctoral experience of each member, summarized in the resumes of the members, which are posted on the EC website [8]. Committee members with advanced professional degrees of medicine, dentistry, or pharmacy were categorized as “clinical,” whereas those with relevant academic degrees in engineering or science were considered as “technical/engineering” or “biological.” In a few instances, members had multiple degrees and/or experiences suggesting that they should be included in two or more categories. The strength of the educational background for each category was assigned a value of 2 if the member had a formal degree or certification in that domain; 1 if modest training was present but below the level expected of a scientist who would otherwise function as an independent professional in that domain; and 0 if no obvious formal training was held in that domain.

The same researchers independently assessed the professional and scholarly attributes of each member under the same domains of clinical, technical/engineering, and biological by examining publications and other information listed in the same publishes resume and then expanding the evaluation using google scholar, researchgate.com, and other relevant search engines. In addition, the types and anatomical targets of devices with which the member had worked were identified to understand further the specializations of each member. Within each domain, a value of 2 was assigned when more than 3 publications or sustained professional activities were found; 1 was assigned when modest activities were reflected by only one or two relevant research products or professional assignments; and 0 was assigned when no professional activities or work products obviously fit that classification. The independent scores were then compared and discussed in order to confirm a final score for the member in each domain, deferring to the highest score given if agreement was not reached easily. Thus, each member’s profile was represented by six values of 0–2, three representing academic background and three representing scholarly profile across the domains.

To illustrate the strength of the panel as a whole in each of the three domains, the values assigned for all members in that domain were summed and then divided by the number of members. This calculated value could range from 0 to 2; a value for a specific domain close to 2 would suggest that the members of that panel had very strong capabilities in that domain, whereas a value close to 0 suggested much weaker levels of expertise.

### Content Analyses of SOs

Content analysis was carried out on the ten (10) published SOs available by February 2023. The extent to which each SO addressed issues in clinical, technical/engineering, and biological domains was scored according to the extent and depth of relevant discussion in that domain. This was not a critique of the scientific correctness of the assessments. A score of 2 was given if text relevant to an area accounted for most of the text, 1 if it accounted for less than 20% of the text, and 0 if no text related to that area could be recognized. The scores for the 10 SOs in each domain were summed and divided by 10. The resultant score was used as an indicator of the amount of attention given by the expert panels to clinical, technical/engineering, and biological issues; values close to 2 suggested that the issues in that domain were strongly considered in the SO, whereas values close to 0 suggested that issues in that domain were given little attention.

## Results

### Educational Background and Professional Experience of Committee Members

The committee for *General and Plastic Surgery and Dentistry* had thirteen members, six of whom were experts in oral and dental therapy and seven in general or plastic surgery. Nine were identified to have a “clinical” academic background only, none an engineering/technical background only, and one a biological background only. The remaining two members had blended academic backgrounds, one with a clinical and biological background and the other with a biological and engineering/technical background. All 13 members had strong clinically oriented professional profiles. Three also had a strong record of publications and ancillary activities in the biological domain and six had modest activities in the biological (3) or technical/engineering (3) domains. Overall scores for each domain related to academic background and professional profile were: Background- clinical, 1.85; biological, 0.15; technical 0.23; Professional Profile- clinical, 2.00; biological, 0.69; technical, 0.23.

The committee for *Orthopedics, Traumatology, Rehabilitation, Rheumatology* had twenty-two members, fourteen of whom were identified as having a “clinical” background only and 3 as having an engineering/technical background only. The remaining five members had blended backgrounds of clinical and engineering /technical education(4) or biological education(1). Eighteen of the 22 members had strong professional profiles in the clinical domain. Of these, two also had strong profiles and six also had modest profiles in the biological domain; two had strong profiles and six had modest profiles in the technical/engineering domain. An additional two had modest clinical profiles but strong profiles in the biological domain (1) or the technical/ engineering domain (1). A single individual had no research publications in any of the domains. Most of the members had expertise related to the hip, knee, or spine. Overall scores for each domain related to academic background and professional profile were: Background- clinical, 1.64; biological, 0.09; technical 0.55; Professional Profile- clinical, 1.73; biological, 0.59; technical, 0.73.

The committee for the *Circulatory System* had 26 members, 22 of whom were identified as having a “clinical” background only and none as having a biological or engineering/technical background only. Four members had a blended background; two had a medical and biological advanced degree and two had a medical and engineering/technical degree. Twenty-three of the 26 members had strong clinical profiles. Of these, one had a strong profile and two had modest profiles in the biological domain, whereas four had modest profiles and one had a strong profile in the technical/engineering domain. Three had modest clinical profiles, of whom one also had a modest profile in the biological domain; the other two had no demonstrable research profile in either of the two other domains. Overall scores for each domain related to academic background and professional profile were: Background- clinical, 2.00; biological, 0.01; technical 0.12; Professional Profile- clinical, 1.88; biological, 0.15; technical, 0.27.

The committee for Neurology had 14 members. Twelve had a medical degree and two of these had an additional PhD; the other two members had a PhD with a technical focus. None had an advanced degree in biological science. Among these with clinical backgrounds, 10 had strong clinical research profiles and two had more modest clinical research profiles; four of these also had strong to modest biological profiles and three had modest technical profiles. The two individuals with a technical background had strong technical research profiles; one also had a strong biological and modest clinical professional profile. Overall scores for each domain related to academic background and professional profile were: Background- clinical, 1.71; biological, 0; technical 0.36; Professional Profile- clinical, 1.64; biological, 0.57; technical, 0.57.

**Figure 1:**
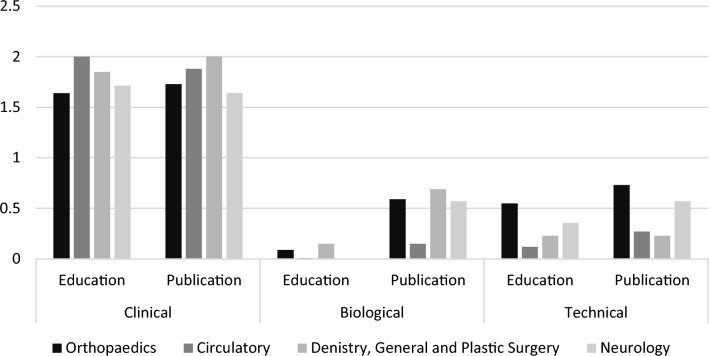
Comparative ranking for expert committees.

The comparative results for these committees are shown in Fig. 1.

### Content of CECP Reviews

All 10 CECPs reports addressed the quantity and strength of the clinical evidence presented to the Notified Body (Table 1). The three CECPs published in 2021 also discussed some aspects of technical/engineering and/or biological evidence. One of these had an extensive discussion related to the strength of the preclinical evidence. Discussions related to preclinical technical and biological considerations were less frequent in the seven CECPs published in 2022. One report from 2022, examining a bioabsorbable stent, mentions in limited detail the toxicology and acceptability of an animal model in preclinical studies. Only three had modest remarks that suggested the need for more information or that supported the recommendations of the Notified Bodies. The other four did not discuss the preclinical technical or biological evidence at all.

**Table 1:** CECP Content Review Rankings

CECP	Clinical	Technical	Biological
2021-000201	2	1	1
2021-000205	1	2	1
2021-000207	2	N	1
2022-000213	2	N	N
2022-000216	2	N	1
2022-000222	2	1	N
2022-000225	2	N	1
2022-000227	2	N	N
2022-000232	2	N	N
2022-000235	2	N	N
Avg.	1.95	0.40	0.50

## Discussion

The role of expert panels appears to have changed in the short time since they were described by MDR regulations in 2017. The MDR identifies that the panels were formed to provide a scientific opinion on certain types of devices based on:

(i) the novelty of the device or of the related clinical procedure involved, and the possible major clinical or health impact thereof; (ii) a significantly adverse change in the benefit-risk profile of a specific category or group of devices due to scientifically valid health concerns in respect of components or source material or in respect of the impact on health in the case of failure of the device. Chapter 2. 5.1c EU MDR [1]

This role was further clarified by the JRC in MDCG 2020-13, a document designed to guide the work of the panels with a Clinical Evaluation Assessment Report template. That template identified the importance of conducting a thorough examination of the clinical trials and data submitted by an applicant company to the Notified Body. However, it also recognized that the evaluation should extend beyond clinical issues to considerations of risk assessment and management more broadly. Panels were encouraged to consider if “the results of the manufacturer's risk management supported the use of non-clinical testing methods” and if there was “sufficient information regarding this interaction available from sources other than clinical data” [9].

The transfer of the expert committee program to the EMA appears to coincide with a change in mandate from the broader focus on scientific, technical, and clinical independent review to the more specific evaluation of clinical device performance, mostly derived from clinical trials. As stated in the Commission Staff Working Document SWD, “The purpose of the Clinical Evaluation Consultation Procedure (CECP) is the provision of independent scientific opinions by expert panels designated pursuant to Commission Implementing Decision (EU) 2019/13962 on the NB’s clinical evaluation assessment report (CEAR) based on the manufacturer’s clinical evidence” [10]. Confusion, therefore, exists whether the broader role that was initially assigned to the panels can and will be carried out given the composition and direction given to the panels currently.

It seems clear from the results presented here that the expert panels are well-equipped to carry out clinical evaluations. Most panel members were, by any standard, highly qualified in relevant clinical specialties. Their expertise was also apparent in their thoughtful scientific opinions related to the work of the Notified Bodies to evaluate clinical trials and literature reviews. What seems less clear is whether a clinical evaluation of this type is sufficient to address more comprehensively the risks of novel and complex devices. All of the expert panels characterized here had only a few members with the engineering or biological expertise needed to evaluate, for example, operational vulnerabilities and toxicological threats related to device design, biomaterials, and software. The skewed distribution of members with clinical compared to technical backgrounds seems surprising given that the relatively broad published eligibility criteria for committee members. The rules for these expert committees allow for the assignment of temporary members to a review [11]. In the published reviews, however, no evidence is available to identify whether supplementary reviewers were utilized or a pool of experts has yet to be identified outside of the listed members. If more such experts were included in the expert panels, they might broaden the singular focus on clinical testing to assess risk–benefit more holistically.

These additional considerations are important because technical and biological shortcomings are often the root cause of problems with quality and reliability for medical devices. For example, the highly publicized safety problems associated with metal-to-metal hip implants and breast implants, which are most commonly considered to have instigated some revisions of the medical device regulations, arose from problems of product design or substandard materials that might have been understood through engineering or toxicological testing rather than clinical trials. In the case of metal hip implants, a technical understanding of nano-micro metal particle toxicology and tissue response were critical skill sets [12]. In that of ruptured breast implants, understanding the impact of sub-grade silicone in vivo, including the risk of cancer or lymphoma, played an important role to characterize risk/benefit impact, critical under the EU MDR [13]. Unlike drug trials, in which adverse events typically present early, device problems are often associated with design or manufacturing challenges that can present after months. Device trials by themselves are often insufficient to characterize fully the durability or reliability of the device under consideration. Given the importance for all aspects of review, the mandate for EXPAMED, whether broadly or narrowly defined to include or exclude considerations of performance beyond the clinical domain, needs to be clear to all stakeholders.

The clinical focus of the SOs examined here is encouraged not only by the composition of the panels but also by the types of materials with which they are provided to do their work. The information given to the committees now appears to include only the Clinical Evaluation Assessment Reports (CEARs) developed by the Notified Body and the clinical summary documents (clinical evaluation plan and report, post-market clinical follow-up plan and report) available from the manufacturer. Further, the panels are given a template for their written SOs that solicits their opinions about the strength of the clinical evidence but not the other factors that might prove to shift the benefit-risk profile. Panels asked only about clinical evaluation are unlikely to provide a more holistic view of risk that takes into account other vulnerabilities. Nonetheless, a few panels, mostly prior to 2022, did identify or question technical or biocompatibility risks. These explicitly identified concerns related to preclinical evaluation such as biocompatibility or battery performance but indicated that they lacked sufficient information to assess these potential risks. The work of the committee might be improved if a mechanism were available to provide fuller information about the preclinical analyses upon request of the panel members.

An independent clinical review is not wrong in principle. Nevertheless, the legislative purpose and appropriateness of expectations should be clear and transparent. If the aim of the program is to restrict benefit-risk evaluations to the clinical domain, then the committees appear well designed for success. If, however, the intent is broader, panel activities could be strengthened by increasing the diversity of experts appointed to the panels or by assigning appropriate ad hoc members or consultants to the committees according to the specifics of the device under consideration. There is an opportunity to better define the intent and the measures of success for the implementation of the EXPAMED committees.

## Conclusion

Results in this study describe expert panels under the CECP system that have strong capabilities to evaluate clinical evidence as part of the benefit-risk assessment of novel and high-risk devices. If a singular focus on clinical evidence is to be the specific role of the committees going forward, then the program seems poised to be successful. If, however, the panels are expected to evaluate risk–benefit more broadly, then the program may fall short by failing to examine the adequacy of all types of evidence typically needed for such determinations. Too strong a focus on clinical investigations and literature may distract from the assessment of technical and biological risks that are common to novel devices and are often best understood by other types of preclinical testing. Efforts to broaden the scope and membership of the panels may be important in future when the expert panels will almost certainly be presented with novel devices whose major risks may be related, for example, to novel biomaterials or software designs. Finally, additional transparency is needed on the availability and use of ad hoc experts when reviews are conducted and the responses of Notified Bodies in response to those reviews.

### Limitations and Delimitations

This examination of the CECP program was conducted early in its implementation. Thus, the conclusions may be limited by the relatively small number of committee reports that are available to date, developed by only four of the expert panels. The scope of the study was delimited to considerations of the match between the expert panel system and the original roles defined for the panels. Not considered were the quality and appropriateness of the work that was produced to critique the adequacy of judgments made by the panels.

## Data Availability

The data in this research is based on a review of publicly available publications available at: https://health.ec.europa.eu/medical-devices-expert-panels/experts/list-opinions-providedunder-cecp_en (Accessed 19 March 2024).
